# Study of Prevalence, Severity and Risk Factors of Periodontal Disease in a Portuguese Population

**DOI:** 10.3390/jcm11133728

**Published:** 2022-06-28

**Authors:** Marta Relvas, Paula López-Jarana, Luis Monteiro, José Júlio Pacheco, Ana Cristina Braga, Filomena Salazar

**Affiliations:** 1 Medicine and Oral Surgery Service, University Institute of Health Sciences (IUCS), CESPU, 4585-116 Gandra, Portugal; marta.relvas@iucs.cespu.pt (M.R.); paula.jarana@iucs.cespu.pt (P.L.-J.); julio.pacheco@iucs.cespu.pt (J.J.P.); filomena.salazar@cespu.pt (F.S.); 2 Oral Pathology and Rehabilitation Research Unit (UNIPRO), IUCS, CESPU, 4585-116 Gandra, Portugal; 3 Algoritmi Centre, School of Engineering, University of Minho, 4800-058 Guimarães, Portugal; acb@dps.uminho.pt

**Keywords:** periodontal disease, prevalence of, new classification, oral hygiene

## Abstract

Periodontal disease is a common worldwide oral inflammation/infection affecting tissues that surround and support teeth. This study aims to evaluate the prevalence, extent and severity of periodontal diseases and its risk factors, according to the most recent periodontal classification, in an adult population of Northern Portugal. This observational study collected data from clinical records of patients who attended the University Clinic of Gandra between April 2021 and April 2022. Of a total of 941 patients included, 457 (48.6%) had periodontitis, 253 (26.9%) had gingivitis and the remaining 231 (24.5%) were healthy patients. The prevalence of stage III severe periodontitis was 51.2%, more prevalent in males, and in the age group of 61–70 years. Gingivitis was more prevalent in females, and in the age group of 31–40 years; in both diseases, the most prevalent extension was the generalized one. Using a binary logistic regression, we observe a significant relation of the risk of periodontitis with age (*p* = 0.019; OR 1.033; 95% CI 1.005–1.062), tooth brushing (*p* = 0.002; OR 0.25; 95% CI 0.105–0.599) and dental flossing (*p* = 0.015; OR 0.63; 95% CI 0.09–0.768). This study revealed a high prevalence of periodontitis. Increased age, lack of tooth brushing and flossing were identified as potential risk factors for periodontitis in the investigated Portuguese population.

## 1. Introduction

Periodontal disease, which includes gingivitis and periodontitis, is a common oral inflammation/infection affecting tissues that surround and support teeth [1]. Gingivitis is characterized by bleeding and gingival enlargement and, if not treated, is prone to periodontitis involving loss of periodontal insertion and bone sustainability [2]. These are common causes of teeth loss, which can compromise mastication, aesthetics, self-confidence and quality of life of patients [3]. In addition, periodontal disease is associated with other common systemic conditions such as diabetes, cardiovascular disease, adverse pregnancy outcomes, rheumatoid arthritis and chronic obstructive pulmonary disease [4,5].

According to the Global Burden of Disease Study of 2016, periodontal disease was the 11th most prevalent condition in the world [6] with a prevalence ranging from 20% to 50% [7].

However, very few data provide a comprehensive assessment of the periodontal status of the Portuguese population. A single national epidemiological study was carried out, in 2015, by the *Direção Geral da Saúde* using the Community Periodontal Index of Treatment Needs (CPITN) reporting periodontal disease frequencies of 10.8% for adults and 15.3% for elderly individuals [8].

These results contrasted (especially taking into account the geographic proximity), with the last Spanish national periodontal investigation, where 38.4% of individuals had periodontal pockets, as with other developed countries, where the prevalence ranged from 51% to 88.3% in countries (regions) such as the USA, Italy (Turim), Norway (Troms) or Germany (Pomerânia) [9,10,11].

A global workshop organized jointly by the European Federation of Periodontology (EFP) and American Academy of Periodontology (AAP) in Chicago in 2017 developed case definitions for periodontal procedures to facilitate the uniformity of the data collection around the world and also to avoid disparities related with different assessments of periodontal variables. However, probably due to the fact that the publication of this new consensus is still very recent, there are still limited data from epidemiological studies that adopt these diagnostic criteria in Europe and specially in Portugal [12].

For this reason, it is mandatory to carry out epidemiological studies on the prevalence of periodontal disease in accordance with the recent definitions that will allow a more comprehensive understanding of the current periodontal status of the Portuguese population and evaluation of the associated risks, contributing to an effective comparison with rest of the data worldwide [4].

The aim of this study is to analyze the prevalence, extension and severity of periodontal disease, according to the 2017 Chicago Workshop, in a population of the north of Portugal. The secondary objective is to assess potential indicators of the risk of periodontal disease in this population.

## 2. Materials and Methods

This was an observational study which analyzed patients attending to the Dental Clinic appointments of the University Clinic of Instituto Universitário de Ciências da Saúde (IUCS), (CESPU, Gandra, Portugal), between April 2021 and April 2022. The study was submitted and approved by the ethics commission of the Instituto Universitário de Ciências da Saúde, with reference CE/IUCS/CESPU-05/21 and performed according to the declaration of Helsinki. The populations of the study were patients residing in the North of Portugal area, which corresponded to the PT 11 Nomenclature of Territorial Units for Statistics (NUTS) regions, with over 3.6 million inhabitants, the NUTSII region with the greatest population in Portugal. The Dental Clinic of the Instituto Universitário de Ciências da Saúde CESPU (Gandra, Portugal) receives patient from the functional urban areas composed by Guimarães, a medium-sized area with 180,000 inhabitants, a metropolitan area of Porto with 1,270,000 inhabitants, Póvoa de Varzim with 60,000 inhabitants, and Braga with 250,000 inhabitants. To achieve an estimate of the prevalence of periodontitis in the population, with a margin of error of 3.0%, for a confidence level of 95%, it was necessary to examine a minimum of 941 individuals, based on previously reported national prevalence data of 10.8% and 15.3% for adults and the elderly, respectively. The required sample was stratified according to the number of adult subjects (age group 18 to 64 years) and elderly (65 years or more).

### 2.1. Research Participants

The patient recruitment was done by inviting them to participate at the main hall-room of the dental clinic of the Instituto Universitário de Ciências da Saúde (IUCS). The patients were carefully informed by oral and written explanation about the objective and procedures of the study. The patients who accepted to participate in the study were asked to sign an informed consent form and fill out a questionnaire before the periodontal examination. The inclusion criteria were that the patients be residents of the north of Portugal, of ages between 18 and 85 years. The exclusion criteria were as follows: pregnant women and subjects with current or previous history of oral and maxillofacial cancer, radiation and oral mucosal pathology also involving periodontium having undergone periodontal treatment in less than 6 months, undertaking oncological treatment, taking bone-related medication and patients aged less than 18 years or more than 85 years. The recorded data from anamneses included: gender, age, smoking habits (nonsmoker; smoker of up to 10 cigarettes per day; smoker of more than 10 cigarettes per day), diagnosis of diabetes mellitus (DM), oral hygiene habits such as frequency of toothbrushing (no brushing, once a day, twice a day, three times a day or more), and use of dental floss and interdental brush. The periodontal clinical data included: number of absent teeth; number of teeth with mobility, pocket depth (PD), measured as distance from the gingival free-margin from the bottom of the pocket; gingival recession (REC) as the distance from the enamel–cement junction (CEJ) to the free gingival margin, (showing a negative signal whenever the gingival margin is located coronary at the (CEJ); clinical attachment loss (CAL); plaque index (IP) and bleeding on probing (BoP). This was registered in six locations per tooth (mesio-vestibular, vestibular, disto-vestibular, mesio-lingual, lingual and disto-lingual), using a CPITN 15 Hu-Friedy Europe Periodontal Probe, Rotterdam, the Netherlands. Wisdom teeth were excluded for analysis.

### 2.2. Cases Definition

Gingivitis and periodontitis were defined according to the new consensus of the AAP/EFP [13]. Gingivitis was considered to be when the total percentage of bleeding in probe (BoP) was ≥10%. Periodontitis was considered when interproximal CAL (clinical attachment loss) was detected in two or more interproximal sites not adjacent or there was an interproximal CAL of 3mm or more, non-vestibular or lingual/palatal, for ≥2 teeth. The periodontitis stage was defined according to the severity of the extension [13]. For staging, interdental CALs of loss of 1–2 mm, 3–4 and ≥5 mm were considered mild (stage I), moderate (stage II) and severe (stage III)/very severe (stage IV), respectively [13]. The presence of complex factors of stage modifiers implies that the stage is altered for a higher stage. Stage IV was differentiated from stage III by modifying factors including: greater than or equal to 5 teeth lost due to periodontal injury (PD); presence of masticatory dysfunction; secondary occlusal trauma; severe alveolar bone defect, <20 remnant teeth. Extension of periodontal disease was classified as localized (<30% two teeth involved) or generalized (>30% two teeth involved).

### 2.3. Measurement Reliability and Reproducibility of Examinators

Two examiners previously trained by an experienced senior periodontic specialist (FS, MR) were calibrated in order to join the measurement criteria, using 10 volunteers on 2 different days, 48 h apart. The calibration was achieved by the measurements of the same random volunteers by the two examiners, registering the grade of reproducibility. The intra-examiner coefficients of correlation (CCI) were 0.97 and 0.98 for CAL and PD and the inter-examiner CCI were 0.99 and 0.98 for CAL and PD 3.7.

### 2.4. Statistical Analysis

The data were analyzed using IBM SPSS Statistics (version 27.0). A descriptive analysis was performed, evaluating quantitative and qualitative variables through graphs and tables. Furthermore, a chi-square test was carried out, to evaluate the relationship between the periodontal diseases and the different risk factors, such as age, brushing habits, use of dental floss, use of interdental brush, tobacco habits and diagnosis of diabetes mellitus (DM).

To assess the risk of occurrence of periodontitis with the presence of the different risk factors evaluated, the methodology of binary logistic regression was used, using a stepwise variable selection technique. The evaluation of the predictive capacity was carried out using the analysis through the Receiver Operating Characteristic (ROC) curve, namely through the discriminant capacity index and area under the curve (AUC). The significance level used was 5%.

## 3. Results

### 3.1. Demographic Data

From an initial number of 1207 patients, 266 were excluded, resulting in a total sample of 941 patients, including 496 females and 445 males (1.1:1) aged between 18 and 85 years (M = 49, SD = 16.7).

### 3.2. Outcomes

Of the included patients, 457 (48.6%) (245 males, 212 females) had periodontitis (95% CI: 45.4–51.8%), 253 (26.9%) had gingivitis (95% CI: 24.1–29.8%) and the remaining 231 (24.5%) were healthy patients (95% CI: 21.9–27.4%). With regard to the severity of periodontitis, the most frequent stage was stage III (51.2%) (95% CI: 46.6–55.8%), followed by stage IV (30.4%) (95% CI: 26.3–34.7%). Only 6.1% of the patients had stage I (95% CI: 4.2–8.6%) and 12.3% stage II (95% CI: 9.5–15.5% CI).

**Figure 1:**
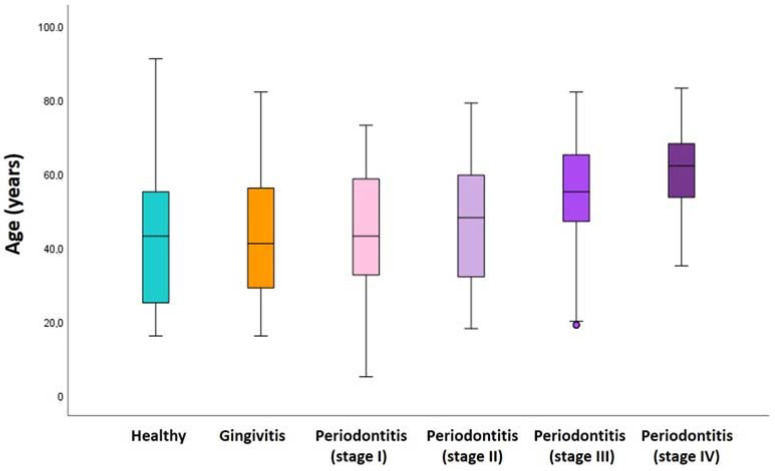
Density plot exhibiting the distribution of periodontal conditions over the age range.

The extent of periodontitis was classified as generalized in 66.5% of patients and as localized in 33.5% patients. Of the 26.9% patients with gingivitis, 92.1% had a generalized extension and only 7.9% a localized extension. Periodontitis was more prevalent in males (55.1%) than in females (42.7%), while gingivitis was more prevalent in females (31.3%) than in males (22%) (X^2^ (2, N = 941) = 15.66, *p* < 0.001). In a total of 457 patients with periodontitis, the most prevalent age group was 61- to 70-years-old (28.2%), followed by the 51 to 60 year group (24.5%). A significant relation (X^2^ (12, N = 941) = 144.495; *p* < 0.001) was observed between age cohort and the appearance of periodontal disease, especially for older patients (Figure 1).

In patients with periodontitis, we observed more smoker patients than nonsmoker patients, but without statistical significance (X^2^ (6, N = 941) = 9.219; *p* = 0.162). From 90 heavy smoker patients (>10 cigarettes/day), 47 (52.2%) were diagnosed with periodontal disease, while from 114 smokers (≤10 cigarettes/day) 46 (40.4%) had periodontal disease (Table 1).

A statistically significant relation was found between the diagnosis of DM and periodontal disease (X^2^ (2, N = 940) = 33.499; *p* < 0.001), where from the 87 diabetic patients, 78.2% (68) presented with periodontal disease compared to 853 non-diabetic patients where this percentage was 45.6% (389). The distribution of presence of DM by age is listed in Table S1.

Data on routine dental-hygiene-instrument use were only available for 204 patients who answered with the information required. The most common brushing frequency were twice per day, corresponding to an observed periodontal disease for 41.7% of the patients of this group. Periodontal disease was related with the tooth brushing because it appeared to be associated with the absence or lower frequency of tooth brushing (X^2^ (6, N = 204) = 23.843; *p* < 0.001). About 166 patients did not use dental floss and 194 did not use interdental brushes. A statistically significant relation (X^2^ (2, N = 204) = 18.110; *p* < 0.001) was found between periodontal disease and dental-floss use, but not with interdental brushes (X^2^ (2, N = 204) = 1.791; *p* = 0.408).

Table 2 shows the distribution of the mean and standard deviation of PD, CAL, REC, PD 4–5 mm, PD ≥6 mm, CAL 4–5 mm and CAL ≥6 mm, along with PI and BoP, according to the gender and age. The results show that males have higher values than females, except for the PD 4–5 mm which was higher in females than in males, and that mean values increase with age. Relative to the plaque index the mean was 46.45%, higher for men than women (47.62%) vs. 45.4%). Bleeding on probing was higher for women (27.15%) than men (25.05%).

### 3.3. Relation between Periodontal Disease and Risk Factors

The results of the logistic regression with the stepwise selection (with a probability for entry = 0.05 and for removal = 0.1) including the variables with a potential relation with the presence of periodontal disease (age, sex, smoking, brushing frequency, dental flossing, interdental brushing and presence of diabetes mellitus) are presented in Table 3.

Thus, the estimated logit model translates into:

$$g\left(x_{i}\right)=B_{0}+B_{1}x_{1}+\cdots +B_{p}x_{p}$$

$$g\left(x_{i}\right)=1.857+0.033\times Age-1.385\times Brushing-1.334\times Dental\;floss+2.119\times Diabetes$$

In terms of estimated probability, the model would be:

$${\hat{\pi }}_{i}=\frac{\exp\left(g\left(x_{i}\right)\right)}{1+\exp\left(g\left(x_{i}\right)\right)}$$

The obtained model is statistically significant, χ^2^(4) = 42.372, *p* < 0.001. The predictive capacity of the model found was evaluated through the area under the ROC curve (AUC) for the estimated probabilities, having obtained AUC = 0.801, which indicates that the model predicts correctly in about 80.1% of cases. In the view of this, we obtained an OR for:

- Age: B = 0.033 (for 10 years, B = 0.033 × 10 = 0.33) > OR = 1.39, it means that for every 10 years increase in age, the risk of periodontitis increases about 1.39 times;
- Tooth brushing: B = −1.385 > OR = 0.250, means that the increase in brushing frequency enhances the non-appearance of periodontitis, favoring a healthy state;
- Flossing: B = −1.334 > OR = 0.263, means that the use of dental floss favors the non-appearance of periodontitis by about 3.8 times (1/0.263);
- Diabetes: B = 2.119 > OR = 8.325, means that diabetics are about 8.3 times more likely to have periodontitis.

## 4. Discussion

The objective of our study on an adult population in the north of Portugal was to determine the prevalence, extension, severity and risk factor associated with periodontal disease on Portugal, using the new classification of periodontal disease [14,15]. Our study found a prevalence of gingivitis of 26.9%, (31.3% for women versus 22% for men) a lower value compared to another Egyptian study about plaque-induced gingivitis where the prevalence was 100% [16]. The higher prevalence for women was similar to a study of Caribbean adults [17] and different from the Egyptian result, where women were less affected [16] (42.2%). The results of this study also showed a prevalence of stage III periodontitis, frequently between 61 to 70 years old and for the male gender. Regarding the extent of periodontal diseases, there was a higher prevalence of generalized extension. In Portugal, to date, there are few studies that comprehensively assess the periodontal status of the Portuguese population. However, in 2019, a study was carried out in the Lisbon Metropolitan Area [12], where mild periodontitis (stage I) was the most prevalent (59.9%), followed by severe periodontitis (stages III and IV) (24%), with moderate (stage II) being the least prevalent (22.2%). Compared with the present study, it can be stated that the populations of northern Portugal have a high prevalence of severe periodontitis (51.2%) and a lower prevalence of mild periodontitis (6.1%) [12]. With regard to clinical variables, a mean PD of 2.29 mm was obtained, which was higher in males than in females. These results were similar to those found in a study carried out in 2018 in a Portuguese subpopulation in Lisbon [15]. Regarding the variation in the mean PD and mean CAL with age, in the present study, both increased. These results are in line with the study carried out in Japan; however, these averages were higher in the latter [18]. For the variables CAL 4–5 mm and mean CAL, they increased with age both in the present study and in the study carried out in the metropolitan area of Lisbon [12]. The variable CAL 4–5 mm was higher in the Lisbon study, while the mean CAL was higher in the present study. This may be due to the fact that the average age in the Lisbon metropolitan study [12] is much higher than in our sample (61 versus 49).

The BoP mean in the present study was 26.16%, higher than that observed in the Lisbon metropolitan study (14.8%) [12] and lower than the observed in studies in Japan [18] (31.0%) and Norway [11] (30.0%). The BoP in these last two studies was higher in females than in males, as observed also in the present study. Regarding PI, the average observed was 46.45%, twice that observed in the study in Lisbon [15] (23.2%) and higher than in the study in Norway [11] (44.2%). In the study conducted in Japan, the mean PI was the highest of the cited studies (59.5%) [18].

The prevalence of smokers in the present study was 21.7%, much lower than the prevalence obtained in the study of the Lisbon subpopulation in 2018 (66%) [19]. It was found that although periodontitis was markedly higher in smokers, the relationship between the two was not statistically significant. Our results contrast with other studies where there was a relationship between smokers and periodontitis [3,10,20]. Epidemiological studies have shown that diabetes mellitus is associated with an increased risk of developing periodontitis, particularly if poorly controlled [21,22]. The present study demonstrated that the occurrence of periodontitis is related to the presence of diabetes mellitus. There are several studies that support this relationship [12,18,21,22]. In fact, in the new consensus classification of periodontal diseases; it establishes diabetes as a modifier for the progression of periodontitis through levels of glycosylated hemoglobin (HbA1c) [23]. Diabetes increases the risk of periodontitis (especially if poorly controlled), and evidence suggests that advanced periodontitis also compromises glycemic control [3].With regard to tooth brushing, in the present study (taking into consideration that this sample was reduced to 204 patients), most patients had a brushing frequency of twice a day (71%), higher than that found in the Lisbon metropolitan study [12] (52.6%) and India [19] (21.1%) and lower than that found in studies from Japan [18] (87.5%), Lisbon [15] (77.3%) and Norway [11] (71.9%). Furthermore, a statistically significant association has been shown between the occurrence of periodontitis and the absence or infrequency of brushings [11,18,19]. Regarding the use of dental floss, in the present study 24.4% reported using this auxiliary means of hygiene, while in the Lisbon metropolitan study only 17.4% used it [12]. A higher value was found in the study of a Portuguese subpopulation from Lisbon, being 34.8% [15]. In addition, a statistically significant association has been shown between the occurrence of periodontitis and not using dental floss [19]. Regarding the use of a brush, there was no association between the occurrence of periodontitis and its use. In the analyzed studies, few or almost none evaluated this parameter. In the study from Japan, 44.5% reported using these dental devices [18].

We acknowledge some limitations in our study including the potential bias in population representation (e.g., due to being a clinic-based population study) or a lack of information from some patients in some variables such hygiene habits, or even some social-demographic variables. Nevertheless, as positive points of this study, using a rigorous methodology, the most recent classification of the AAP/EFP was used, allowing future comparability with other studies worldwide. The results of this study provide new data that will enable appropriate public oral health programs for population-based preventive actions even in the post-pandemic period [24]. These results show that a comprehensive national oral program, with greater accessibility for all and with an emphasis on periodontal diseases, is imperative. A national study is urgently needed to compare these data with national studies from other countries, to help design population awareness programs in this area and to track risk groups highlighted by the model developed in this study, namely the elderly, diabetics and smokers.

## 5. Conclusions

In the present adult population of northern Portugal, the prevalence of periodontal health was 24.5%, gingivitis was 26.9% and periodontitis was 48.6%. There was a high prevalence of severe and very severe periodontitis (81.6%), mainly among the elderly. Regarding the extent of periodontal diseases, the most frequent prevalence in both gingivitis and periodontitis was generalized (92.1% and 66.5%, respectively). There was a relationship between age and the occurrence of periodontitis, the latter being associated with more advanced age groups. Periodontal disease showed a relationship also with diabetes mellitus in the sense that the occurrence of periodontitis is associated with the presence of the disease. It is necessary to increase the population’s awareness of periodontitis, its signs, symptoms and consequences for general health.

## Figures and Tables

**Table 1:** Summary for distribution of categorical variables for each level of stage of disease.

		No Disease	Gingivitis	Stage I	Stage II	Stage III	Stage IV	Total
**Gender**	**Male**	102 (44.2%)	98 (38.7%)	11 (39.3%)	26 (46.4%)	131 (56.0%)	77 (55.4%)	445 (47.3%)
	**Female**	129 (55.8%)	155 (61.3%)	17 (60.7%)	30 (53.6%)	103 (44.0%)	62 (44.6%)	496 (52.7%)
**Age**	**18–30**	75 (32.5%)	66 (26.1%)	5 (17.9%)	11 (19.6%)	15 (6.4%)	0 (0%)	172 (18.3%)
	**31–40**	33 (14.3%)	55 (21.7%)	8 (28.6%)	13 (23.2%)	18 (7.7%)	6 (4.3%)	133 (14.1%)
	**41–50**	43 (18.6%)	47 (18.6%)	7 (25%)	10 (17.9%)	51 (21.8%)	21 (15.1%)	179 (19.0%)
	**51–60**	42 (18.2%)	41 (16.2%)	1 (3.6%)	10 (17.9%)	66 (28.2%)	34 (24.5%)	194 (20.6%)
	**61–70**	29 (12.6%)	29 (11.5%)	5 (17.9%)	7 (12.5%)	62 (26.5%)	55 (39.6%)	187 (19.9%)
	**71–80**	3 (1.3%)	14 (5.5%)	2 (7.1%)	5 (8.9%)	20 (8.5%)	20 (14.4%)	64 (6.8%)
	**+80**	6 (2.6%)	1 (0.4%)	0 (0%)	0 (0%)	2 (0.9%)	3 (2.2%)	12 (1.3%)
**Tobacco Habits**	**Non-smokers**	174 (75.3%)	198 (78.3%)	21 (75%)	42 (75.0%)	193 (82.5%)	104 (74.8%)	732 (77.8%)
	**Ex-smokers**	1 (0.4%)	0 (0%)	1 (3.6%)	0 (0%)	1 (0.4%)	2 (1.4%)	5 (0.5%)
	**Former smokers** **(<10 c/d)**	30 (13%)	38 (15%)	3 (10.7%)	8 (14.3%)	21 (9%)	14 (10.1%)	114 (12.1%)
	**Current smokers** **(>10 c/d)**	26 (11.3%)	17 (6.7%)	3 (10.7%)	6 (10.7%)	19 (8.1%)	19 (13.7%)	90 (9.6%)
**Diabetes**	**No**	221 (96.1%)	243 (96.%)	25 (89.3%)	54 (96.4%)	205 (87.6%)	105 (75.5%)	853 (90.7%)
	**Yes**	9 (3.9%)	10 (4%)	3 (10.7%)	2 (3.6%)	29 (12.4%)	34 (24.5%)	87 (9.3%)
**Tooth Brushing Frequency**	**Do not Brush**	0 (0%)	1 (1.7%)	0 (0%)	0 (0%)	5 (11.1%)	3 (12.5%)	9 (4.4%)
	**Brush 1 per day**	4 (8.9%)	11 (19%)	4 (28.6%)	8 (44.4%)	13 (28.9%)	10 (41.7%)	50 (24.5%)
	**Brush 2 per day**	35 (77.8%)	42 (72.4%)	10 (71.4%)	10 (55.6%)	24 (53.3%)	11 (45.8%)	132 (64.7%)
	**Brush 3 or more per day**	6 (13.3%)	4 (6.9%)	0 (0%)	0 (0%)	3 (6.7%)	0 (0%)	13 (6.4%)
**Dental Flossing**	**No**	31 (68.9%)	41 (70.7%)	13 (92.9%)	16 (88.9%)	41 (91.1%)	24 (100%)	166 (81.4%)
	**Yes**	14 (31.1%)	17 (29.3%)	1 (7.1%)	2 (11.1%)	4 (8.9%)	0 (0%)	38 (18.6%)
**Interdental Brushing**	**No**	42 (93.3%)	57 (98.3%)	14 (100%)	18 (100%)	39 (86.7%)	24 (100%)	194 (95.1%)
	**Yes**	3 (6.7%)	1 (1.7%)	0 (0%)	0 (0%)	6 (13.3%)	0 (0%)	10 (4.9%)

**Table 2:** Summary of distribution of categorical variables for periodontal parameters related to age.

		PD	PD 4–5 mm (%)	PD ≥6 mm (%)	CAL	CAL 4–5 mm (%)	CAL ≥6 mm (%)	BoP	PI
**Sex**	**M**	2.4 (0.83) [1.07–7.9]	12.11 (11.45) [0.13–54.1]	8.52 (11.07) [0–68.93]	3.53 (1.82) [0–9.36]	14.94 (13.85) [0.52–66]	17.29 (20.54) [0.52–88.88]	25.05 (24.27) [0–100]	47.62 (32.01) [0–100]
	**F**	2.18 (.73) [1.05–5.93]	12.56 (12.5) [0.55–57.97]	7.02 (9.35) [0–64.58]	3.02 (1.8) [0–7.98]	11.92 (10.8) [0.52–91.02]	12.76 (16.96) [0.53–81.15]	27.15 (24.6) [0–100]	45.4 (29.63) [0–100]
**Age Mean (SD)** **[95% CI]**	**18–30**	1.85 (0.42) [1.78–1.91]	4.79 (6.67) [3.03–6.54]	2.94 (4.71) [–0.5–5.94]	1.55 (1.58) [1.31–1.79]	3.26 (3.63) [2.26–4.27]	2.78 (3.14) [1.31–4.25]	17.58 (18.09) [14.85–20.3]	34.99 (24.43) [31.32–38.67]
	**31–40**	2.07 (0.59) [1.97–2.17]	9.13 (11.2) [6.38–11.88]	5.05 (7.82) [0.72–9.39]	2.27 (1.34) [2.04–2.5]	6.04 (8.56) [3.97–8.11]	3.33 (4.0) [1.89–4.78]	23.92 (21.89) [20.17–27.68]	44.06 (28.75) [39.13–49.0]
	**41–50**	2.33 (0.86) [2.22–2.45]	11.74 (10.94) [9.72–13.76]	8.59 (12.61) [5.27–11.9]	3.41 (1.57) [3.18–3.64]	10.97 (12.25) [8.91–13.03]	11.24 (16.14) [8.02–14.46]	23.93 (22.53) [20.61–27.26]	43.99 (29.35) [39.66–48.32]
	**51–60**	2.43 (0.85) [2.31–2.55]	14.09 (12.8) [11.99–16.19]	8.45 (11.15) [5.98–10.91]	3.89 (1.58) [3.67–4.12]	14.59 (10.82) [12.97–16.21]	15.37 (18.73) [12.16–18.59]	31.37 (26.63) [27.6–35.15]	51.38 (33.16) [46.69–56.08]
	**61–70**	2.55 (0.83) [2.43–2.67]	14.62 (12.29) [12.62–16.63]	8.06 (9.75) [6.04–10.08]	4.31 (1.47) [4.09–4.52]	17.69 (12.79) [15.76–19.61]	20.25 (21.9) [16.66–23.83]	30.94 (27.79) [26.92–34.96]	55.25 (30.86) [50.79–59–71]
	**71–80**	2.53 (0.77) [2.34–2.77]	14.84 (13.68) [11.03–18.65]	7.87 (7.88) [4.76–10.99]	4.18 (1.37) [3.84–4.53]	18.31 (14.59) [14.51–22.21]	17.08 (19.16) [11.74–22.41]	32.41 (25.73) [25.98–38.83]	51.02 (33.99) [42.53–59–51]
	**+80**	2.55 (0.75) [2.07–3.02]	13.78 (14.10) [2.95–24.62]	7.67 (8.56) [5.95–21.29]	5.12 (0.96) [4.51–5.73]	27.1 (13.08) [18.79–35.41]	29.81 (17.23) [18.23–41.38]	15.2 (15.94) [4.99–25.24]	33.39 (39.52) [28.0–58.5]

M, male; F, female; PD, pocket depth; BoP, bleeding on probing; CAL, clinical attachment loss; PI, plaque index.

**Table 3:** Results of binary logistic regression.

Variables	B	SE(B)	W	*p*	OR	95% CI for OR	
						LL	UL
**Age**	0.033	0.014	5.493	0.019	1.033	1.005	1.062
**Tooth brushing**	−1.385	0.445	9.692	0.002	0.250	0.105	0.599
**Dental flossing**	−1.334	0.546	5.972	0.015	0.263	0.090	0.768
**Diabetes mellitus**	2.119	1.152	3.385	0.066	8.325	0.871	79.582
**Constant**	1.857	1.118	2.758	0.097	6.406		

B, estimates for the slope coefficients of the univariate logistic regression model containing only this variable; S.E., estimated standard error for the estimated coefficient; W, Wald statistics; *p*, value associated with the statistical coefficient test; OR, estimated odds ratio; CI, confidence interval of 95% for odds ratio; LL, lower limit; UL, upper limit.

## Data Availability

The data can be accessed by contacting the corresponding author.

## Supplementary Materials

The following supporting information can be downloaded at: https://www.mdpi.com/article/10.3390/jcm11133728/s1, Table S1: Distribution of the presence or absence of systemic diseases (Diabetes mellitus) by age.
