# Supplementary material for: Study of Prevalence, Severity and Risk Factors of Periodontal Disease in a Portuguese Population
Source: J Clin Med. 2022 Jun 28;11(13):3728. doi: 10.3390/jcm11133728 (PMC9267442; doi:10.3390/jcm11133728)
Supplement: Supplementary file 1 [file jcm-11-03728-s001.zip › jcm-1753195-supplementary.pdf]

Suplemmetal Table S1 – Distribution of the presence or absence of systemic diseases (Diabetes mellitus) by age

	**Diabetes mellitus**					
	No		Yes		Total	
	Count	%	Count	%	Count	%
18-30	170	19.9%	2	2.3%	172	18.3%
31-40	132	15.5%	0	0.0%	132	14.0%
41-50	173	20.3%	6	6.9%	179	19.0%
51-60	177	20.8%	17	19.5%	194	20.6%
61-70	148	17.4%	39	44.8%	187	19.9%
71-80	45	5.3%	19	21.8%	64	6.8%
+ 80	8	0.9%	4	4.6%	12	1.3%
Total	853	100.0%	87	100.0%	940	100.0%
